# Improving the Pass-Band Return Loss in Liquid Crystal Dual-Mode Bandpass Filters by Microstrip Patch Reshaping

**DOI:** 10.3390/ma7064524

**Published:** 2014-06-13

**Authors:** Javier Torrecilla, Virginia Urruchi, José Manuel Sánchez-Pena, Noureddine Bennis, Alejandro García, Daniel Segovia

**Affiliations:** 1 Departamento de Tecnología Electrónica, Universidad Carlos III de Madrid, C.\Butarque n° 15, Leganés 28911, Spain; E-Mails: vurruchi@ing.uc3m.es (V.U.); jmpena@ing.uc3m.es (J.M.S.-P.); 2 Institute of Applied Physics, Military University of Technology, Kaliskiego 2, Warsaw 00-908, Poland; E-Mail: nbennis@wat.edu.pl; 3 Departamento de Teoría de la Señal y Comunicaciones, Universidad Carlos III de Madrid, C.\Butarque n° 15, Leganés 28911, Spain; E-Mails: alamperez@tsc.uc3m.es (A.G.); dani@tsc.uc3m.es (D.S.)

**Keywords:** liquid crystals, tunable devices, dual-mode filters, inverted-microstrip structure, microwave frequencies

## Abstract

In this paper, the design and experimental characterization of a tunable microstrip bandpass filter based on liquid crystal technology are presented. A reshaped microstrip dual-mode filter structure has been used in order to improve the device performance. Specifically, the aim is to increase the pass-band return loss of the filter by narrowing the filter bandwidth. Simulations confirm the improvement of using this new structure, achieving a pass-band return loss increase of 1.5 dB at least. Because of the anisotropic properties of LC molecules, a filter central frequency shift from 4.688 GHz to 5.045 GHz, which means a relative tuning range of 7.3%, is measured when an external AC voltage from 0 V_rms_ to 15 V_rms_ is applied to the device.

## 1. Introduction

During the last two decades, liquid crystals (LC) have become a promising approach for the design of tunable devices at microwave frequencies. The anisotropy of the molecules of these materials allows LC electrical properties to depend on their molecules orientation, which can be changed by applying an external electric field. Concretely, the LC permittivity can be varied between two extreme values, ε_r⊥_ and ε_r‖_, as a function of the applied voltage. This is the reason which permits LC-based devices to be voltage-controlled. Some examples of tunable devices based on LC technology at microwave frequencies in the bibliography are capacitors [1], antennas [2], filters [3], *etc*.

Moreover, the advantages of using LC in size, cost or power consumption [4] compared to other studied technologies for designing tunable microwave devices, make these materials very suitable for this purpose. For example, varactor diodes need high voltages, up to 30 V, in order to achieve large tuning ranges for frequencies higher than 1 GHz [5], while devices based on ferrite technology [6] require a magnetic field for tuning, which leads to problems in terms of size and power consumption. Therefore, the improvement of the existing LC-based prototypes and the development of new LC tunable devices at these frequencies are interesting challenges to be faced in the immediate future.

Filters are very important devices at microwave telecommunication systems, for example, in satellite communications, due to their ability of selecting (bandpass filters) or rejecting (bandstop or notch filters) bands of frequencies. Furthermore, the demand on the development of tunable filters has increased during the last years. In this work, a tunable LC-based band-pass filter with microstrip geometry is presented. A reshaping of a dual-mode square patch geometry reported in previous works has been proposed as the microstrip filter. This new patch structure is expected to improve the filter performance, increasing the pass-band return loss. A high return loss is desirable in a filter frequency response because it involves a lower pass-band filter insertion loss variation. The design, manufacture and experimental results of the device are detailed in this paper.

## 2. Liquid Crystal Bandpass Filter Design

Since dual-mode technology for bandpass filters was introduced by Wolff in 1972 [7], these filters have been widely used in microstrip technology because of their advantages, specifically in size reduction. This is a critical issue in LC-based devices, since a size reduction involves a decrease of the amount of used LC and, thus, a lower manufacturing cost. Dual-mode operation consists of a resonant circuit where two electromagnetic degenerated modes are excited due to the presence of some kind of perturbation in the microstrip structure. Therefore, a two-pole filter can be obtained with a single resonator, which implies a significant size reduction. For implementing dual-mode filters in microstrip technology, different topologies have been used, such as square-loops [8] or T-shaped stubs [9].

**Figure 1:**
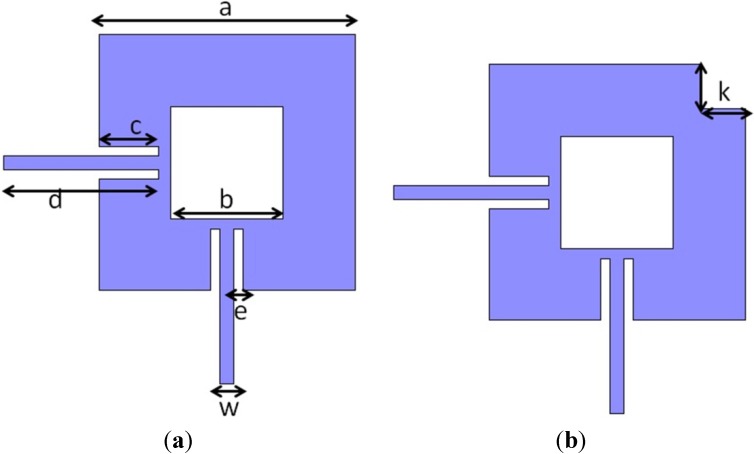
Geometry of a microstrip square patch resonator with a central square notch (dual-mode). (**a**) Without any additional cut; (**b**) With a square cut on a specific corner.

The topology shown in Figure 1a is a dual-mode filter structure developed by S. Li *et al.* [10] achieving a good performance [11]. It consists of a microstrip metallic square patch with a central square notch and two perpendicular feed lines for exciting the two degenerated modes. In this work, a reshaping of the patch of this structure has been proposed. The modification consists of making a square cut of size k etched on the patch, as it is shown in Figure 1b. This new microstrip patch is expected to improve the passband return loss (S_11_ parameter) and to narrow the filter bandwidth. 

The value of the filter central frequency (*f*_c_) for the structure presented in Figure 1 is a function of the structure dimensions and the effective permittivity (ε_eff_). It is especially sensible to variations in the *b* dimension, while filter bandwidth depends on the *a*, *c* and *e* dimensions [10]. Thus, for fixed dimensions of the structure, *f*_c_ can be varied as ε_eff_ is modified. In addition, permittivity depends on the substrate materials of the device, so a suitable choice of the substrate materials has been the strategy to achieve the device tunability. In this work, LC is used as the dielectric substrate of the microstrip line in the device. As LC permittivity changes between two extreme values, ε_r__⊥_ and ε_r__‖_, by applying voltage, the filter central frequency is also voltage-controlled. Therefore, the use of LC as dielectric substrate is the reason which allows the filter to be tunable.

**Figure 2:**
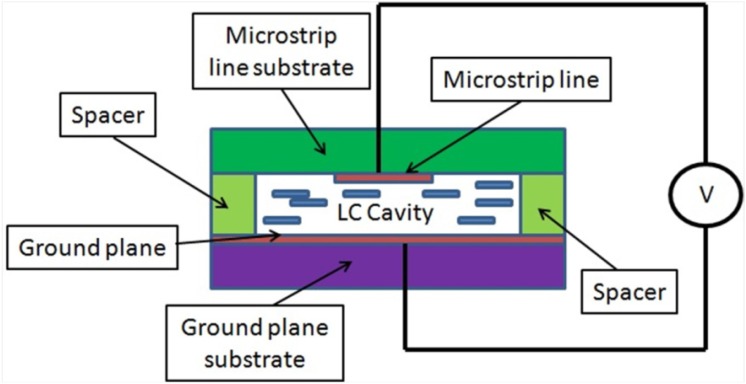
An inverted-microstrip structure for a LC bandpass filter. The detail of the parts of the filter is shown. Note that figure is not drawn to scale.

LC is a fluid material, so it needs to be confined in a cavity inside the device which will be afterwards sealed. For this reason, the filter is implemented by using an inverted-microstrip structure, as shown in Figure 2. This structure has been already reported in other LC-based devices [12]. The LC cavity is designed of 0.25-mm thick and delimited by the spacers; the dielectric material Taconic TLX-08 (ε_r_ = 2.55) is employed for them. This material is also used as the substrate that supports the microstrip line with 0.8-mm thick.

Before the fabrication of the device and the filling with the LC, several electromagnetic simulations by using the software Ansoft HFSS have been run for optimizing the filter dimensions. Initially, the LC cavity has been considered to be empty in the simulations, that is, ε_r_ = 1. As it is recalled in Section 3, LC permittivity extreme values are unknown at microwave frequencies. The dimensions of the patch have been chosen by comparing the spectral response of the filter with a conventional dual-mode patch (Figure 1a) and with the new patch with a square cut (Figure 1b). The results of the patch dimensions for the topology presented in Figure 1a is solved for a filter central frequency nearby 7.5 GHz considering empty the LC cavity. These values are summarized in Table 1. The microstrip line width (*w*) has been designed of 0.65 mm in order to obtain input impedance (*Z*_in_) close to 50 Ω. 

**Table 1:** Results for the dimensions of the conventional dual-mode patch. Some concerned parameters are: bandwidth (BW), central frequency (*f*_c_) and input impedance (*Z*_in_).

Dimension	Value	Comments
a	11.6	Changes in BW
b	5	Changes in *f_c_*
c	2.8	Changes in BW
d	10	Changes in BW
e	0.5	Changes in BW
w	0.65	Changes in *Z_in_*

**Figure 3:**
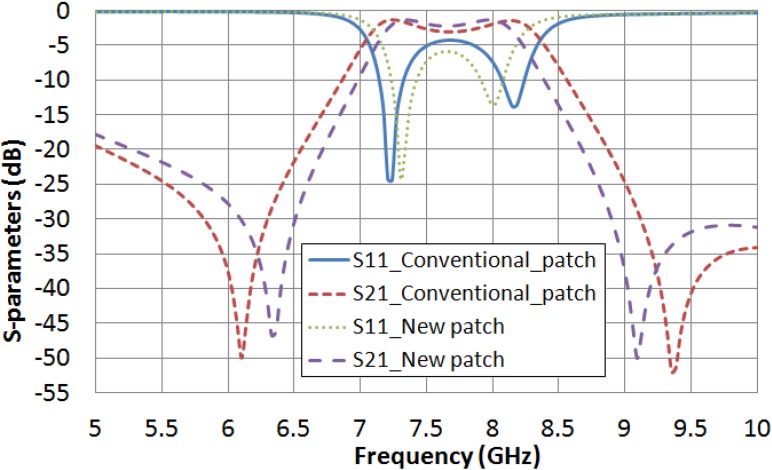
S-parameters obtained in simulation for both filters with a conventional dual-mode patch and with the new dual-mode one with a square cut.

Once the dimensions have been fixed, a square cut with side k is made in the conventional microstrip square patch. The new dual-mode structure (Figure 1b) is simulated for several values of *k*. With *k* = 2 mm a significant band-pass return loss improvement is achieved. Figure 3 shows a comparison between the filter frequency response (parameters S_21_ and S_11_) obtained in simulation for the new dual-mode patch and for the conventional one. Table 2 summarizes the performance of both structures.

**Table 2:** Comparison between the performances of both dual-mode filters.

Structure	Central Frequency (*f*_c_)	Bandwidth (BW)	Return Loss (RL)
With a conventional patch	7.69 GHz	1320 MHz	4.3 dB
With a new patch	7.665 GHz	1050 MHz	5.8 dB

The results for the empty filters suggest that the patch with the new shape improves the pass-band return loss (RL) and narrows the filter bandwidth of the filter. A filter bandwidth reduction of 20.5% is achieved, which leads to obtain a pass-band return loss increase of 1.5 dB.

## 3. Manufacturing of the Device

Once the viability and improvements of the new dual-mode structure have been confirmed with the simulations in an empty device, the device is manufactured and built over an inverted-microstrip structure (Figure 2). As it was described previously, Taconic TLX-08 (Taconic Europe, Ry, Denmark) is used for the spacers and for the microstrip line support. FR4 (ε_r_ = 4.4) is used as the substrate which supports the ground plane and it is not expected to affect the filter behavior.

**Figure 4:**
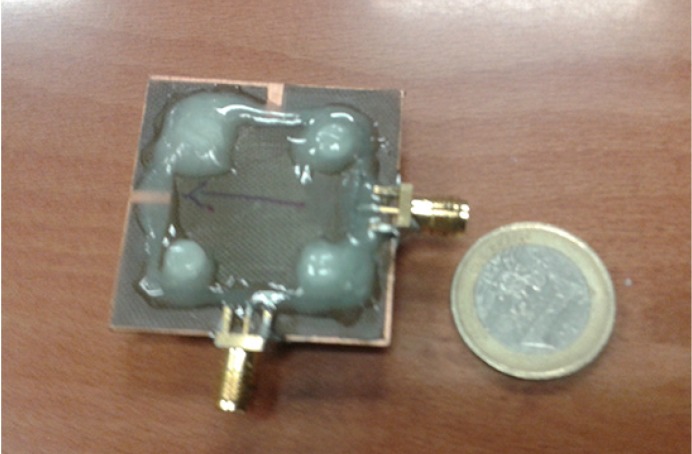
Manufactured LC bandpass filter.

The manufactured device (Figure 4) is filled with the nematic LC Merck MDA-98-1602 (Merck, Darmstadt, Germany), whose properties are, initially unknown at microwave frequencies. It is important to take account that LC are materials employed, specially, for the design of tunable electrooptical devices, so they are not usually tested at microwave frequencies. In the manufacturing process, LC molecules are aligned parallel to the microstrip line by rubbing a thin film of polyimide which acts as an alignment layer. In these conditions, when no voltage is applied, the LC permittivity is minimum, ε_r__⊥_. As voltage is applied, the molecules rotate and, at a saturation voltage value, LC molecules are oriented nearly perpendicular to the microstrip line; at this point, LC permittivity reaches its maximum value, ε_r‖_. The tunability of the filter central frequency is achieved; the LC permittivity increases, while the filter central frequency decreases, as voltage is applied.

## 4. Experimental Results and Discussion

The device S-parameters are measured by using an Agilent 8703B network analyzer (Agilent Technologies, Santa Clara, CA, USA). A sinusoidal AC signal of 1 kHz is used as the voltage for switching the LC. In order to superimpose the LC driving voltage with the microwave signal, a bias-T device is connected between the port 1 of the analyzer and the filter input.

**Figure 5:**
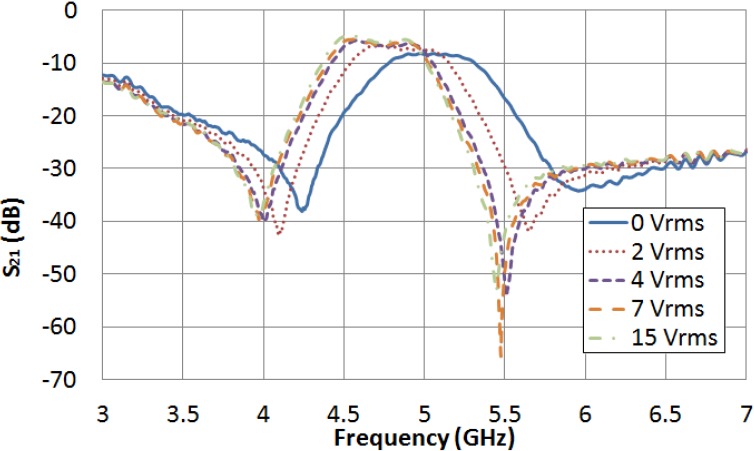
LC driving voltage dependence of the S_21_ parameter for the new bandpass filter.

**Figure 6:**
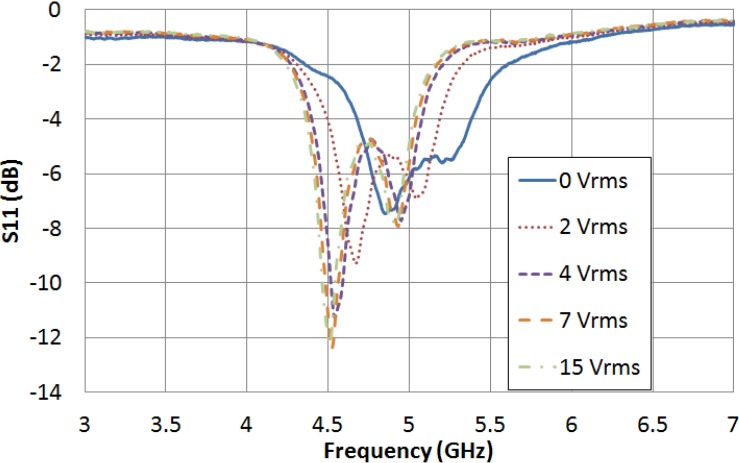
LC driving voltage dependence of the S_11_ parameter for the new bandpass filter.

**Figure 7:**
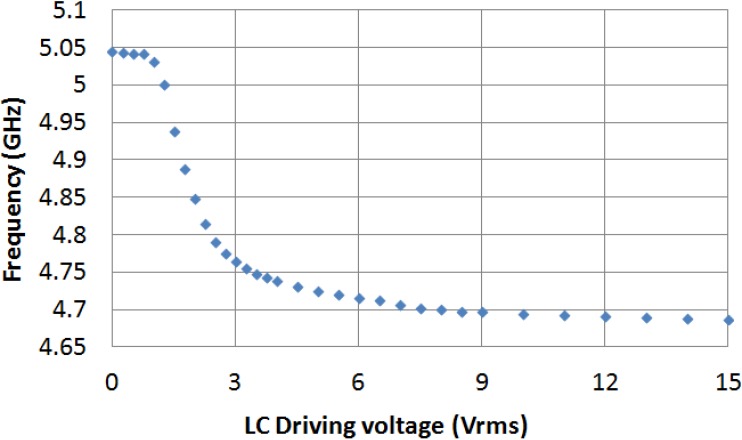
Tunability of the filter central frequency as a function of the LC driving voltage.

The filter is measured for several values of external LC driving voltage between 0 V_rms_ and 15 V_rms_. Figure 5 and Figure 6 graph the frequency dependence of S_21_ and S_11_ parameters, respectively, for different values of applied voltage. In the absence of applied voltage, the filter central frequency reaches its maximum value, 5.045 GHz. As it was expected, this value is clearly below the obtained in simulation when the LC cavity was considered to be empty (7.665 GHz), because LC permittivity is supposed to be greater than 1. As an increasing voltage is applied, the LC permittivity increases, so the filter central frequency decreases. When the LC driving voltage reaches the saturation value (15 V_rms_), the filter central frequency is minimum, 4.688 GHz, because of the fact that the LC permittivity is maximum, ε_‖_. The tunability of the filter central frequency as a function of the LC driving voltage is shown in Figure 7.

A filter central frequency tuning range of 357 MHz is achieved, which means a tuning relative range of 7.3%. Comparison between liquid crystal filters is not an easy task because multiple criteria, for comparing performance of the filters, are available: central frequency, filter bandwidth, number of poles, topology, *etc.* However, not at all comparable between them. Tunable bandpass filters of Table 3 share some characteristics that are comparable between them, independently of the specific structure, configuration and dimensions. Therefore, the new filter we have proposed, with a compact and non-complex structure, has similar performance, in terms of relative tuning range, than filters with the same number of poles [13,14]. But, the performance could be improved even further if high dielectric anisotropy liquid crystals were used [15]. Of course, by increasing the number of poles of the filter [3], leads to higher performance but also to higher filter complexity. 

**Table 3:** Filter performance compared to other LC-based bandpass filters.

LC-based filter	Centroal Frequency (*f*_0_)	Relative Tuning Range	Number of Poles	Comments
Presented work	5 GHz	7.3%	2	Dual-mode filter structure. Merck MDA-98-1602 as used LC
Goelden *et al.* [3]	20 GHz	10%	3	3-pole microstrip filter. A highly anisotropic LC is used
Bernigaud *et al.* [13]	5 GHz	6%	2	DBR filter structure. Merck BL037 as used LC
Missaoui *et al.* [14]	5 GHz	4.8%	2	2-pole DBR structure. Merck LC K15 is used

The filter bandwidth gets narrower as an increasing voltage is applied, while the relative bandwidth, defined as the ratio between the bandwidth and the central frequency, remains constant. The pass-band return loss of the filter slightly gets worse as an increasing voltage is applied. Table 4 shows a summary of the measurements.

**Table 4:** Summary of the experimental measurements of the spectral response for the new LC bandpass filter.

LC Drive Voltage	LC Dielectric Constant (ε)	Central Frequency (*f*_c_)	Bandwidth (BW)	Relative Bandwidth (BW/*f*_c_)	Return Loss (RL)
0 V_rms_	ε_r__⊥_	5.045 GHz	636 MHz	0.126	5.4 dB
15 V_rms_	ε_r‖_	4.688 GHz	568 MHz	0.122	4.9 dB

The variation of the frequency as a function of the applied voltage shown in Figure 7 suggests that the LC director 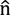, which is the vector that represents the local average orientation of LC molecules in the LC bulk, has an average tilt angle which changes as voltage is applied. For low values of voltage, tilt angle is close to 0°, while it is nearby 90° for high values of voltage. This average tilt angle presents a quasi-linear dependence for intermediate values of the applied voltage, while it tends to saturation for extreme values.

**Figure 8:**
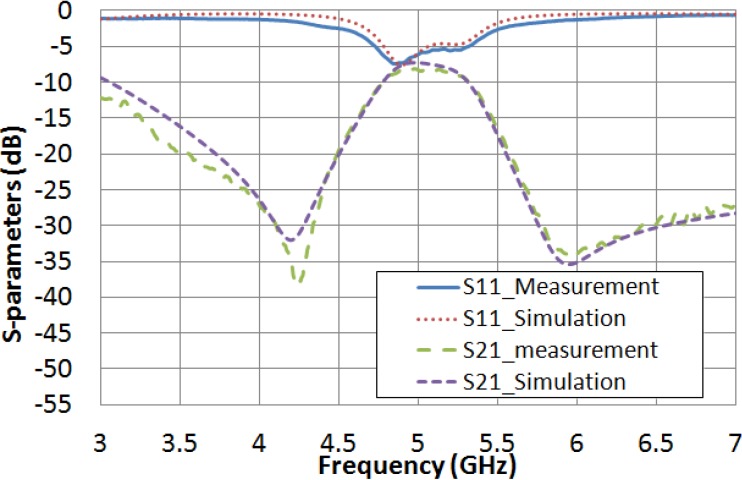
Comparison between the measured frequency response when no voltage is applied and the simulated considering when ε_r_ = 2.62 and tan δ = 0.05.

**Figure 9:**
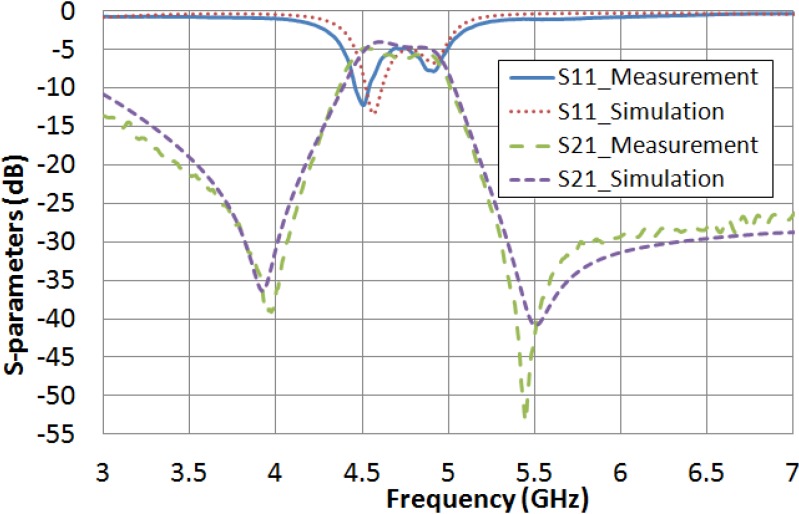
Comparison between the measured frequency response when 15 V_rms_ are applied and the simulated considering when ε_r_ = 3.06 and tan δ = 0.02.

A complementary analysis of the spectral response performance, comparing the simulations and the measurements, has been carried out this time for the filter filled with the LC. Due to LC features are initially unknown at microwave frequencies, an iterative process is programed for fitting both experimental and simulated responses. The routine considers several values for LC permittivity and loss tangent, and keeps the device dimensions invariant. As a result, the software protocol gives rise to a preliminary estimation of the dielectric properties of the LC. Therefore, the extreme values of permittivities, ε_r__⊥_ and ε_r__‖_, and LC loss tangent, tan δ_⊥_ and tan δ_‖_, are inferred. As it is shown in Figure 8, a fairly good agreement between the measured filter frequency response (when no voltage is applied) and the simulated response (with ε_r_ = 2.62 and tan δ = 0.05) is achieved. In the same way, the measured frequency response by applying the saturation voltage value (15 V_rms_) and the simulation response, considering ε_r_ = 3.06 and tan δ = 0.02, are also in reasonable agreement (Figure 9).

Therefore, in a preliminary estimation, it can be inferred that the dielectric properties for the used LC at 5 GHz are the shown in Table 5.

**Table 5:** Preliminary estimation of the dielectric properties extreme values for the LC Merck MDA-98-1602.

Applied LC drive voltage	Estimated LC permittivity (ε)	Estimated LC Loss Tangent (tan δ)
0 V_rms_	ε_r__⊥_ = 2.62	tan δ_⊥_ = 0.05
15 V_rms_	ε_r‖_ = 3.06	tan δ_‖_ = 0.02

Let us note that LC loss tangent estimated values are high. This may be the reason why the filter exhibits high insertion loss, as it is shown in Figure 5.

**Figure 10:**
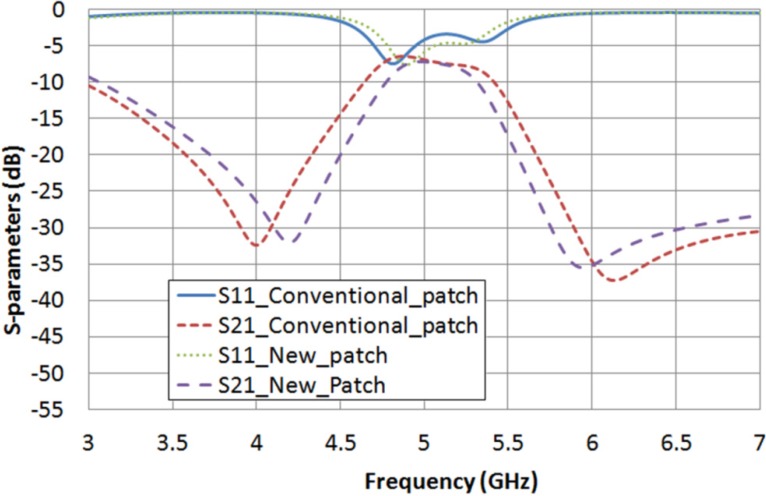
S-parameters obtained in simulation for both structures considering ε_r__⊥_ = 2.62 and tan δ_⊥_ = 0.05.

**Figure 11:**
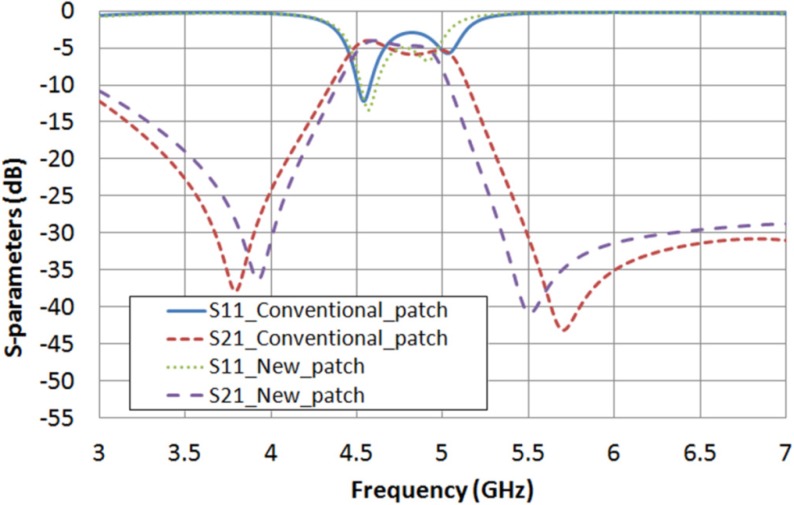
S-parameters obtained in simulation for both structures considering ε_r__‖_ = 3.06 and tan δ_‖_ = 0.02.

With these estimated values, a comparison between the microstrip conventional square patch and the new patch, supposing the cavity filled with LC, can be obtained in simulation for the extreme values of LC permittivity and loss tangent. Figure 10 shows the frequency response of both structures considering ε_r__⊥_ = 2.62 and tan δ_⊥_ = 0.05. In Table 6, the performances of the new square patch structure and the conventional one are summarized. A filter bandwidth narrowing of 14.7% and a pass-band return loss increase of 1.5 dB, which means an increase of 30.6%, are achieved by using the new patch. In the same way, Figure 11 and Table 7 present a comparison between both structures when ε_r__‖_ = 3.06 and tan δ_‖_ = 0.02 are considered, obtaining a filter narrowing of 17.8% and a pass-band return loss improvement of 1.9 dB, which means an improvement of 38.8%.

**Table 6:** Comparison between the performance of both dual-mode filters considering ε_r__⊥_ = 2.62 and tan δ_⊥_ = 0.05.

Structure	Central Frequency (*f*_c_)	Bandwidth (BW)	Return Loss (RL)
With a conventional patch	5.037 GHz	737 MHz	3.4 dB
With a new patch	5.039 GHz	629 MHz	4.9 dB

**Table 7:** Comparison between the performance of both dual-mode filters considering ε_r__‖_ = 3.06 and tan δ_‖_ = 0.02.

Structure	Central Frequency (*f*_c_)	Bandwidth (BW)	Return Loss (RL)
With a conventional patch	4.723 GHz	661 MHz	3 dB
With a new patch	4.671 GHz	543 MHz	4.9 dB

## 5. Conclusions

In this work, a tunable LC-based bandpass filter using dual-mode microstrip technology has been designed and experimentally measured. A reshaping of a square patch dual-mode geometry has been proposed as the microstrip filter. This new structure is expected to achieve a significant pass-band return loss improvement.

Because of LC molecules dielectric anisotropy, a filter central frequency variation from 4.688 GHz to 5.045 GHz is experimentally obtained, which means a relative tuning range of 7.3%. The experimental results have led to a preliminary estimation of the LC permittivity and loss tangent, whose values were initially unknown at microwave frequencies, obtaining ε_r__⊥_ = 2.62 and ε_r__‖_ = 3.06 for the LC permittivity and tan δ_⊥_ = 0.05 and tan δ_‖ _= 0.02. Simulations have been run supposing these estimated values, confirming the improvements of using the new dual-mode structure. 
